# Molecular detection of *Bartonella* species in wild small mammals in western Yunnan Province, China

**DOI:** 10.3389/fvets.2023.1301316

**Published:** 2023-11-21

**Authors:** Yun-Yan Luo, Dan Yu, Hong-Ze Zhang, Zheng-Xiang Liu, Ru-Dan Hong, Mei Hong, Zhi-Qiong Ai, Jun-Jie Zhu, Jia-Xiang Yin

**Affiliations:** ^1^School of Public Health, Dali University, Dali, China; ^2^Yunnan Institute of Endemic Disease Control and Prevention, Dali, China

**Keywords:** *Bartonella*, small mammals, geographical variations, phylogenetic analysis, western Yunnan

## Abstract

**Background:**

Small mammals serve as the main reservoir for *Bartonella* and as a proxy indicator of the potential risk of *Bartonella* transmission from nature to humans. They offer a valuable early warning for human infection. Nevertheless, geographical variations in the impact of the host on the occurrence of *Bartonella* infection are underestimated. This study was designed to investigate the infection characteristics of *Bartonella* and explore its species diversity in wild small mammals in western Yunnan Province, China.

**Methods:**

Wild small mammals were captured from Yulong, Jianchuan, and Lianghe counties in western Yunnan Province between 2015 and 2016. Real-time quantitative PCR (qPCR) was used to detect *Bartonella* infection, and the *Bartonella* species were identified by phylogenetic analysis. The factors associated with *Bartonella* infection in small mammals were analyzed by the Chi-square Test.

**Results:**

The prevalence of *Bartonella* in small mammals was 47.85% (768/1605). Lianghe County had the highest *Bartonella* infection rate, with 56.27% of the samples tested positive, followed by a rate of 50.91% was tested in Yulong County, and 39.97% in Jianchuan County (*p* < 0.001). *Bartonella* was detected positive in a total 25 small mammal species, with infection rates ranging from 2.17% to 100%. *Niviventer fulvescens* had the highest *Bartonella* infection rate. In comparison with the dominant small mammal species, *Eothenomys mileyus* had the lowest *Bartonella* infection rate than that in *Apodemus chevrieri*, *Rattus tanezumi*, and *Apodemus draco* (*p* < 0.001). Male small mammals had a higher infection rate than females (*p* < 0.05). The prevalence of *Bartonella* in small mammals during the summer season was higher compared to the other three seasons (*p* < 0.001). Woodland landscape had the highest *Bartonella* infection rate (*p* < 0.001). *Bartonella rochalimae*, *B. japonica*, *B. tribocorum*, *B. washoensis*, *B. sylvatica*, and *B. rattimassiliensis* were obtained from infected small mammals.

**Conclusion:**

This study showed a high prevalence of *Bartonella* was detected with various *Bartonella* species in small mammals in Yulong, Jianchuan, and Lianghe counties of western Yunnan Province. These findings hold significant scientific clues, providing valuable reference points for further research of *Bartonella* natural foci in Yunnan or other analogues environments.

## 1 Introduction

*Bartonella* is the causative agent of Bartonellosis, a facultative intracellular, fastidious, gram-negative bacterium, typically transmitted by parasites that bite, such as fleas, mites, and ticks (1). Humans and vertebrate animals (small mammals, dogs, pets, etc.) are the main reservoir hosts. Currently, there are 39 *Bartonella* species and three subspecies found in different small mammals, horses, cattle, deer, sheep, birds, canids, felids, marine mammals, and reptiles (source: https://lpsn.dsmz.de/genus/bartonella) (2). Previous studies have reported *Bartonella* positive rates in small mammals of 18% to 78% in European (1, 3–7), 20% to 52% in Asia (8–13), and 9.35% in Senegal (Africa) (14). Additionally, one study showed a 50% infection rate in marsupials in Brazil (15). These findings imply that *Bartonella* infection in small mammals might exhibit high adaptability across different geographic region.

Small mammals, as one of the most abundant species in nature, can harboring various pathogens or viruses. Rodent-borne diseases can easily be transmitted to humans when they come into contact with infected small mammals in nature. Therefore, small mammals can act as effective sentinels of natural disease transmission to humans. Especially rodents, as the main hosts of *Bartonella*, can carry up to 20 *Bartonella* species, but the infection rate is varied by region. Of the 20 *Bartonella* species, *Bartonella henselae*, *B. tribocorum*, *B. rochalimae*, *B. elizabethae*, *B. grahamii*, *B. washoensis*, *B. tamiae*, and *B. vinsonii subsp. arupensis* have been responsible for hazarding human health (5, 9, 16). Moreover, the increasing proximity of human activities to natural environments in recent years has led to greater human exposure to small mammals and vectors, indirectly elevating the risk of *Bartonella* infections among people from the natural environment. Thus, it is imperative to pay sustained attention to detecting *Bartonella* in small mammals in various habitats, which can provide effective scientific clues for the etiology and epidemiological research of *Bartonella*. There have been long-lasting reports of *Bartonella* detection in small mammals in Yunnan Province. In western Yunnan Province, the prevalence of *Bartonella* was 5% in small mammals reported by one study in Yulong county in the past decades (17). In Jianchuan county, *Bartonella* was isolated from blood in small mammals in 2017 with a 55.91% positive rate (18). Yang et al. previously detected *Bartonella* in small mammals in Dehong Dai and Jingpo Autonomous Prefecture (Lianghe county was one of the sample sites) with a 24.86% positive rate. However, there was no prevalence of *Bartonella* in Lianghe county (19). Still, it is insufficiently characterized for host-pathogens association of *Bartonella* in western Yunnan Province (18–21), even though there has an ideal environment for small mammals and vectors to survive. This study aimed to investigate the prevalence of *Bartonella* in small mammals collected from Yulong, Jianchuan, and Lianghe counties. Real-time quantitative PCR (qPCR) was used to detect *Bartonella* in small mammals, and phylogenetic analysis was performed to identify the diversity of *Bartonella* species. Additionally, the study examined the relationship between associated factors and *Bartonella* infection in small mammals. This effort is essential to gain a more profound insight into the complex interactions between *Bartonella*, its host species, and the environment, which in turn can inform strategies for disease prevention and management.

## 2 Materials and methods

### 2.1 Study areas and sampling period

The study was carried out in three counties in western Yunnan Province. Yulong and Jianchuan counties are located in the middle of the Hengduan mountain but are separated by low-lying valleys. Yulong county has a low-latitude highland South Asian monsoon climate. Jianchuan county has a low-latitude highland monsoon climate and is concentrated growing pine forests. Lianghe county is located in the southwestern of the Hengduan Mountain and is characterized by the south subtropical monsoon climate. The three counties could be a potential *Bartonella* natural reservoir because the diverse climates and rich vegetation growths benefit small mammals and ectoparasites to survive and reproduce, contributing to unneglected concerns for the residents and travelers.

The study was carried out in four seasons, including winter (which started from December in 2015), spring (Mar in 2016), summer (July to August in 2016), and autumn (October in 2016).

### 2.2 Small mammal collection and DNA extraction

**Figure 1:**
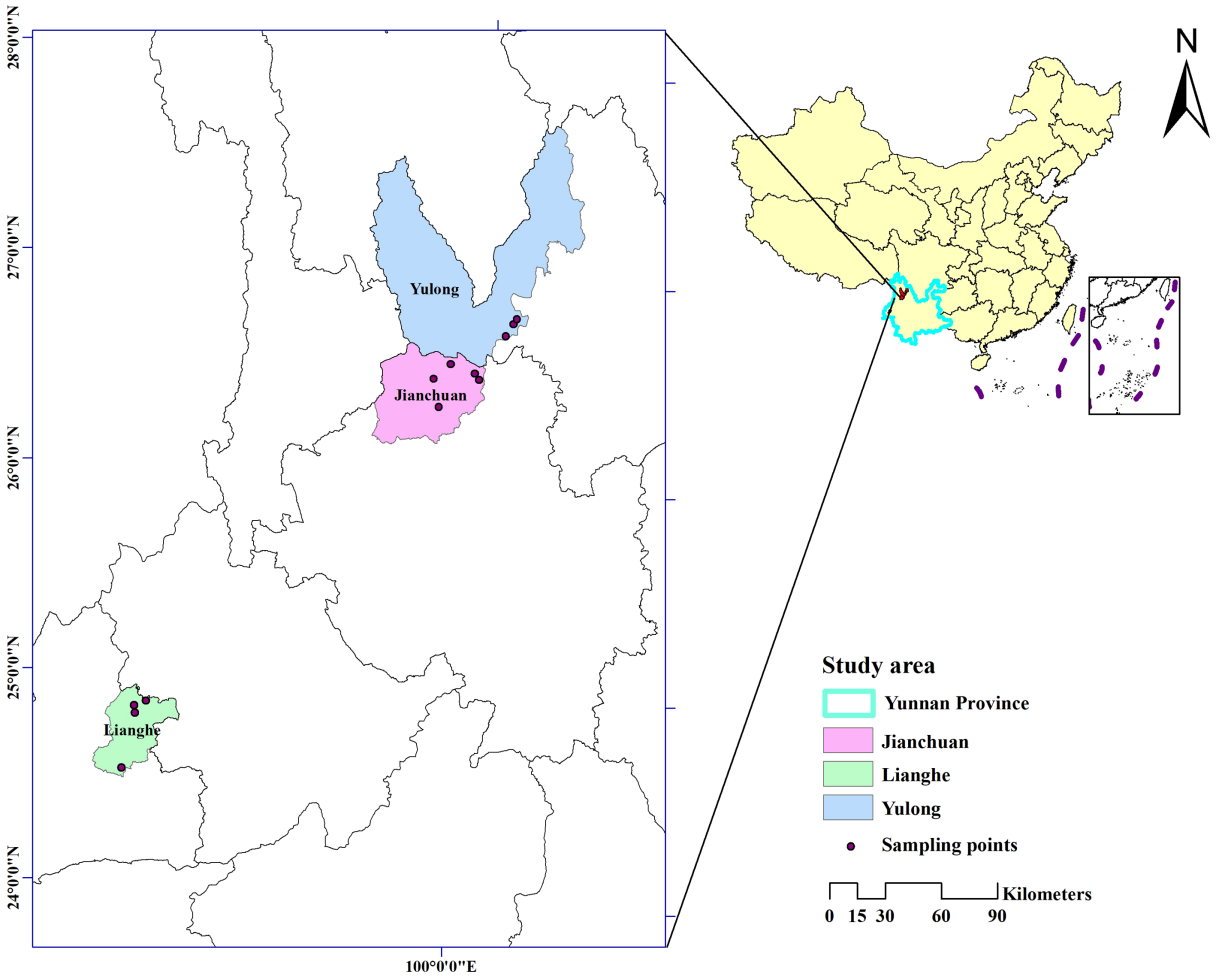
Sampling sites of capturing wild small mammals in the three counties of western Yunnan Province, China. The map was created by ArcGIS 10.2 to show the geographic area of Jianchuan, Yulong, and Lianghe counties in western Yunnan Province.

Small mammals were captured by dead traps (15 × 8 cm). The species were identified by key morphology characteristics of small mammals, spleen or liver tissue of them have been collected under the aseptic condition and stored at −40°C. The type of landscapes was recorded in the fieldwork. In each county, landscapes where small mammals were most likely to be captured were selected based on the annual small mammals monitoring data, including bush, cultivation, and woodland. The details of small mammals captured and tissue collected were described in previous publication (22). The sampling sites of capturing small mammals in the three counties are shown in Figure 1.

Deoxyribonucleic acid (DNA) from the spleen and liver of small mammals was extracted by the BioTeke Whole blood genomic DNA Kit (AU19014-16, BioTeke Corporation, Beijing, China), according to the manufacturer’s instruction and then stored at −40°C until subsequent molecular laboratory.

### 2.3 Real-time PCR

The concentration and purity of the extracted DNA were initially assessed. When the extract concentration was ≥50 μg/mL and A_260_/A_280_ was between 1.8 to 2.1, it was selected as the detection template. Real-time Quantitative Polymerase Chain Reaction (qPCR) was performed to amplify the *ssrA* gene sequence according to previous study (16). One set of primers with 10 μM (AuGCT Biotech, Beijing, China) (F: 5’-GCTATGGTAATAAATGGACAATGAAATAA-3′; R: 5’-GCTTCTGTTGCCAGGTG-3′) and a probe (P: FAM-ACCCCG CTTAAACCTGCGACG-BHQ1) were used under below conditions.

The reaction mixture (20 μL) contains the following components: 0.8 μL each of outer primers F and R, 0.4 μL of the probe, 10 μL of HR qPCR Master Mix (Huirui Biotechnology Co., Ltd., Shanghai, China), and 3 μL of the template. The qPCR condition consisted of pre-denaturation at 94°C for 5 min, denaturation at 94°C for 15 s, and annealing at 60°C for 45 s, followed by 40 cycles. The plasmid standards were used to draw the standard curve. The amplified products were determined according to the standard curve and limit of detection (Cq = 35), if the Cq <35 was positive.

The sequencing of positive samples was generated by conventional PCR according to the above reaction conditions. The amplified products were collected and electrophoresed in 1.5% agarose gel containing Gelview (BioTeke Corporation, Beijing, China) and visualized under the Gel imaging system (G: BOX F3, Syngene, American). The amplified products (269 bp) were confirmed as *Bartonella* positive. Following 10%–15% of the positive samples from each county, which included positive samples from different areas and landscapes whenever possible, were randomly selected to sequence in both directions (Sangon Biotech, Shanghai, China).

### 2.4 Phylogenetic analysis

Sequences of successfully detected were edited and trimmed by DNASTAR (*7.1 version*). Reference complete or partial sequences encoding *ssrA* of *Bartonella* were retrieved from GenBank by the Blast program of the National Center for Biotechnology Information.^1^ Sample sequences were aligned with reference sequences using Sequence distance in Meglign of DNASTAR. Phylogenetic analysis was performed with the Clustal W protocol (default parameters) by Mega software (*7.0 version*). Phylogenetic trees were constructed by the Neighbor-joining method after 1,000 bootstrapped replicates.

### 2.5 Statistical analysis

Demographic, geographic, and laboratory parameters were recorded in Epidata. Wild small mammals were classified as dominant species (>10%) and other species (≤ 10%) according to the constituent ratio. The *Bartonella* infection rate in wild small mammals was calculated by wild small mammal samples infected with *Bartonella* over the number of wild small mammal DNA successfully extracted.

The number of positive were summarized and counted across different species, genus, area, and season using the “tidyverse” package in R software (*4.0.2 version*). The *Bartonella* infection in different genera, species, county, and season were compared using Chi-square Test. *p* values less than 0.05 were considered statistical significance.

### 2.6 Ethics approval

The study has been approved the Medical Ethics Committee of Dali University (no. MECDU-201507-21). The small mammals captured were allowed and no painful.

## 3 Results

### 3.1 Species of wild small mammals and *Bartonella* detection

A total of 1,605 wild small mammal specimens were taken across the three counties (648 in Jianchuan county, 550 in Yulong county, and 407 in Lianghe county), covering 30 different species. There are 22 species of *Rodentia*, 7 species of *Insectivora*, and one species of *Scandentia*. In total, *Apodemus chevrieri* was the dominant species, which accounted for 29.35% (471/1605), followed by *Eothenomys miletus* was 19.00% (305/1605) and *Apodemus draco* was 9.78% (157/1605). In Yulong county, the dominant species were *A. chevrieri* (33.64%), *E. miletus* (16.55%), and *A. draco* (16.00%). In Jianchuan county, the dominant species were *A. chevrieri* (44.14%), *E. miletus* (31.94%), and *A. draco* (10.65%). In Lianghe county, *Rattus tanezumi* (24.57%) was the dominant species, followed by *Rattus rattus* (19.90%), *Mus pahari* (10.81%), and *Niviventer fulvescens* (10.07%). The distribution of wild small mammals across the three counties is shown in Table 1.

**Table 1:** Prevalence of *Bartonella* in wild small mammals captured from the three counties of western Yunnan Province [positive/*n* (%)].

Species	Yulong	Jianchuan	Lianghe	Total
*Apodemus chevrieri*	122/185(65.95)	133/286 (46.5)	0	255/471 (54.14)
*Apodemus draco*	58/88 (65.91)	26/69 (37.68)	0	84/157 (53.5)
*Apodemus latronum*	35/52 (67.31)	10/18 (55.56)	0	45/70 (64.29)
*Anourosorex squamipes*	0	1/1 (100)	19/24 (79.17)	20/25 (80)
*Bandicota indica*	0	0	1/1 (100)	1/1 (100)
*Berylmys bowersi*	0	0	1/9 (11.11)	1/9 (11.11)
*Crocidura attenuate*	0/5 (0)	1/11 (9.09)	4/6 (66.67)	5/22 (22.73)
*Crocidura dracula*	2/6 (33.33)	1/3 (33.33)	3/3 (100)	6/12 (50)
*Collosciurus erythraeus*	0/1 (0)	0	0	0/1 (0)
*Dremomys pernyi*	10/20 (50)	0/1 (0)	0	10/21 (47.62)
*Eothenomys mileyus*	35/91 (38.46)	77/207 (37.2)	3/7 (42.86)	115/305 (37.7)
*Eothenomys proditor*	6/39 (15.38)	0	0	6/39 (15.38)
*Eothenomys eleusis*	0	0	7/9 (77.78)	7/9 (77.78)
*Eothenomys melanogaster*	0	1/2 (50)	0	1/2 (50)
*Hylomys suillus*	0	0	9/28 (32.14)	9/28 (32.14)
*Mus pahari*	0	0	24/44 (54.55)	24/44 (54.55)
*Micromys minutus*	0	0/6 (0)	0	0/6 (0)
*Niviventer andersoni*	1/3 (33.33)	4/6 (66.67)	2/2 (100)	7/11 (63.64)
*Niviventer confucianus*	5/26 (19.23)	4/9 (44.44)	1/2 (50)	10/37 (27.03)
*Niviventer fulvescens*	0	0	35/41 (85.37)	35/41 (85.37)
*Rattus nitidus*	1/1 (100)	0	1/14 (7.14)	2/15 (13.33)
*Rattus tanezumi*	1/2 (50)	0/2 (0)	57/100 (57)	58/104 (55.77)
*Rattus norvegicus*	0	1/4 (25)	0	1/4 (25)
*Rattus rattus*	0	0/1 (0)	44/81 (54.32)	44/82 (53.66)
*Sciurotamias forresti*	0/1 (0)	0	0	0/1 (0)
*Sorex minutes*	0/2 (0)	0	0	0/2 (0)
*Soriculus leucops*	3/4 (75)	0	0	3/4 (75)
*Suncus murinus*	0	0/3 (0)	17/32 (53.13)	17/35 (48.57)
*Tupaia belangeri*	0/23 (0)	0/19 (0)	1/4 (25)	1/46 (2.17)
*Vernaya fulva*	1/1 (100)	0	0	1/1 (100)
Total	280/550 (50.91)	259/648 (39.97)	229/407 (56.27)	768/1605 (47.85)

**Figure 2:**
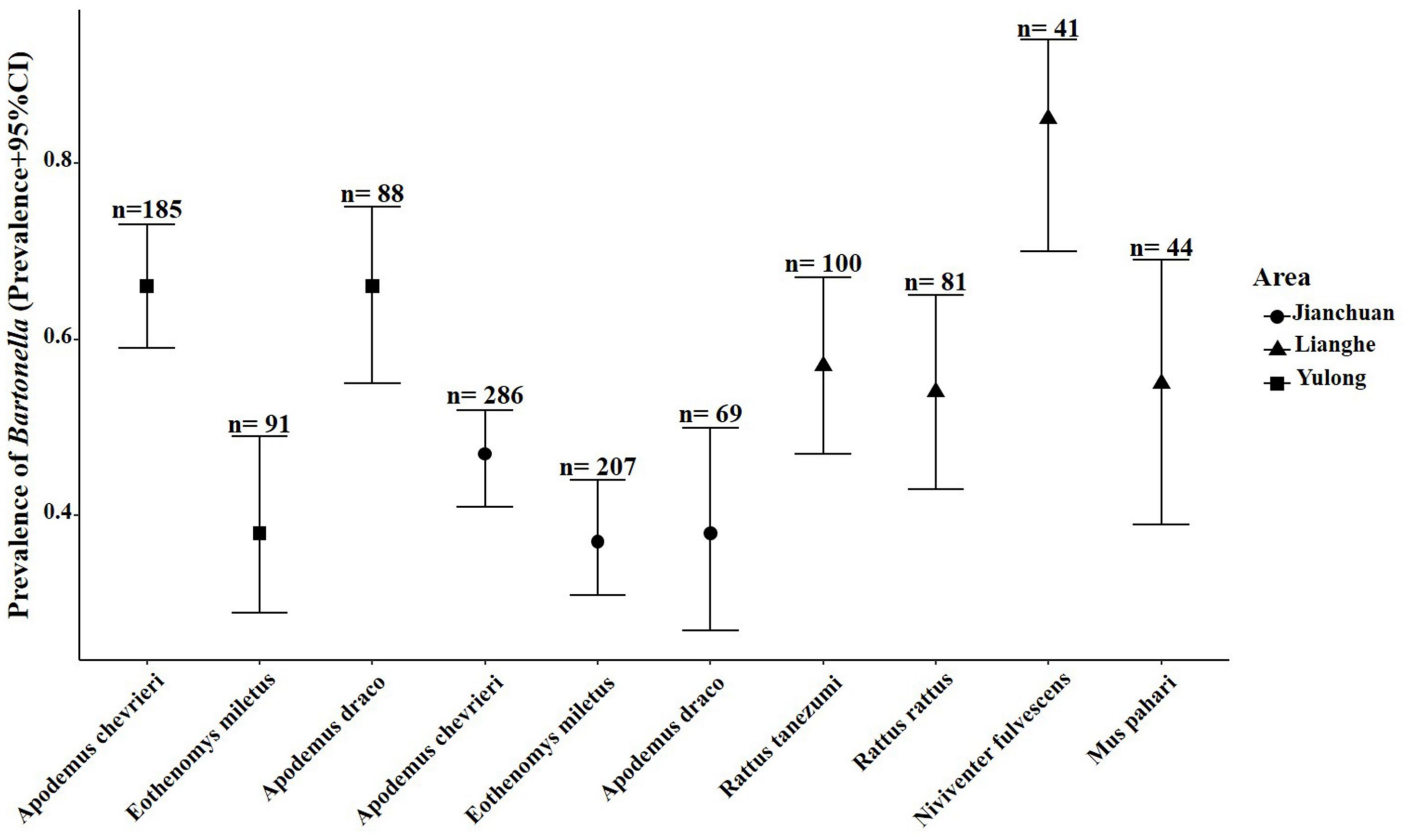
Prevalence of *Bartonella* in the dominant small mammal species in the three counties of western Yunnan Province. Circle represented the prevalence of *Bartonella* in the dominant small mammal species in Jianchuan county, triangle represented the prevalence of *Bartonella* in the dominant small mammal species in Lianghe county, square represented the *Bartonella* positive samples in Yulong county.

Twenty-five various small mammal species were shown positive for *Bartonella* detection. The overall prevalence of *Bartonella* in wild small mammals was 47.85% (768/1605). The result of partial samples of qPCR and agarose gel electrophoresis are shown in Supplementary Figures S1, S2. The *Bartonella* was detected varied from 2.17 to 100% by different species. The prevalence of *Bartonella* was highest in *N. fulvescens* (85.37%, 35/41) and was lowest in *Tupaia belangeri* (2.17%, 1/46). Although *Eothenomys eleusis*, *Soriculus leucops*, *Vernaya fulva*, and *Bandicota indica* had a high prevalence (>60%), the sample size was too small to represent. Seven small mammal species were captured in all three counties, and *Bartonella* was detected in four of them, including *Crocidura dracula*, *E. mileyus*, *Niviventer andersoni*, and *Niviventer confucianus*, with *E. mileyus* had the highest infection rate (37.7%, 115/305), followed by *N. confucianus* (27.03%, 10/37). The infection rates of *C. dracula* and *N. andersoni* were more than 50%, but the sample size was too small to represent. Among the dominant small mammal species across the different counties, *A. chevrieri* had a prevalence of 65.95% (*n* = 185), followed by *A. draco* at 65.91% (*n* = 88), and *E. mileyus* at 38.46% (*n* = 91) in Yulong county. In Jianchuan county, *A. chevrieri* had a prevalence of 46.50% (*n* = 286), followed by *A. draco* at 37.68% (*n* = 69), and *E. mileyus* at 37.20% (*n* = 207). However, in Lianghe, *N. fulvescens* had a prevalence of 85.37% (*n* = 41), followed by *M. pahari* at 54.55% (*n* = 44), *R. tanezumi* at 57.00% (*n* = 100), and *R. rattus* at 54.32% (*n* = 81), the prevalence of *Bartonella* in the dominant small mammal species across different areas is shown in Figure 2.

Eighteen small mammal species were captured in all four seasons, and *Bartonella* was detected in 14 of these species. Among the four seasons, summer exhibited the highest infection rate for *Bartonella* in small mammals (56.52%, 208/368), followed by spring (47.63%, 171/359) and autumn (44.99%, 211/469), while winter had the lowest rate (43.52%, 178/409). *A. chevrieri*, *A. draco* had a higher infection rate in spring and summer, *E. mileyus* had a higher infection rate in summer and winter. In addition, *R. rattus* had higher *Bartonella* infection in autumn and winter (Shown in Table 2). A total of 18 genera of small mammals were captured, of which 14 genera were infected *Bartonella*. The genus of *Anourosorex* had the highest *Bartonella* prevalence (80.00%, 20/25), followed by the genus of *Niviventer* (58.43%, 52/89) and *Apodemus* (55.01%, 384/698). The *Soriculus* genus had 75% *Bartonella* prevalence, but the sample was too small to represent. The prevalence of *Bartonella* distribution of small mammal species and small mammal genera by regions or season are shown in Tables 1, 2.

**Table 2:** Prevalence of *Bartonella* in wild small mammals captured across four seasons in the three counties of western Yunnan Province [positive/*n* (%)].

Genus	Species	Spring	Summer	Autumn	Winter	Total
*Anourosorex*	*Anourosorex squamipes*	5/8 (62.5)	3/3 (100)	8/9 (88.89)	4/5 (80)	20/25 (80)
*Apodemus*	*Apodemus chevrieri*	42/68 (61.76)	82/134 (61.19)	84/166 (50.6)	47/103 (45.63)	255/471 (54.14)
	*Apodemus draco*	29/45 (64.44)	22/32 (68.75)	12/35 (34.29)	21/45 (46.67)	84/157 (53.5)
	*Apodemus latronum*	8/17 (47.06)	11/12 (91.67)	19/30 (63.33)	7/11 (63.64)	45/70 (64.29)
*Bandicota*	*Bandicota indica*	0	1/1 (100)	0	0	1/1 (100)
*Berylmys*	*Berylmys bowersi*	0/3 (0)	0/1 (0)	1/3 (33.33)	0/2 (0)	1/9 (11.11)
*Collosciurus*	*Collosciurus erythraeus*	0	0	0/1 (0)	0	0/1 (0)
*Crocidura*	*Crocidura attenuate*	4/6 (66.67)	0/2 (0)	1/12 (8.33)	0/2 (0)	5/22 (22.73)
	*Crocidura dracula*	1/1 (100)	2/3 (66.67)	2/6 (33.33)	1/2 (50)	6/12 (50)
*Dremomys*	*Dremomys pernyi*	5/10 (50)	0/2 (0)	0	5/9 (55.56)	10/21 (47.62)
*Eothenomys*	*Eothenomys mileyus*	27/90 (30)	31/71 (43.66)	25/73 (34.25)	32/71 (45.07)	115/305 (37.7)
	*Eothenomys proditor*	3/11 (27.27)	1/4 (25)	1/2 (50)	1/22 (4.55)	6/39 (15.38)
	*Eothenomys eleusis*	0	2/4 (50)	5/5 (100)	0	7/9 (77.78)
	*Eothenomys melanogaster*	0	1/2 (50)	0	0	1/2 (50)
*Hylomys*	*Hylomys suillus*	1/4 (25)	2/6 (33.33)	2/8 (25)	4/10 (40)	9/28 (32.14)
*Micromys*	*Micromys minutus*	0/2 (0)	0/1 (0)	0/3 (0)	0	0/6 (0)
*Mus*	*Mus pahari*	10/15 (66.67)	9/12 (75)	4/7 (57.14)	1/10(10)	24/44 (54.55)
*Niviventer*	*Niviventer andersoni*	0/1 (0)	2/3 (66.67)	3/5 (60)	2/2 (100)	7/11 (63.64)
	*Niviventer confucianus*	3/9 (33.33)	3/12 (25)	1/6 (16.67)	3/10 (30)	10/37 (27.03)
	*Niviventer fulvescens*	12/12 (100)	8/8 (100)	7/10 (70)	8/11 (72.73)	35/41 (85.37)
*Rattus*	*Rattus nitidus*	0/8 (0)	0	1/5 (20)	1/2 (50)	2/15 (13.33)
	*Rattus norvegicus*	1/1 (100)	0/3 (0)	0	0	1/4 (25)
	*Rattus rattus*	8/11 (72.73)	6/10 (60)	11/30 (36.67)	19/31 (61.29)	44/82 (53.66)
	*Rattus tanezumi*	11/19 (57.89)	15/17 (88.24)	16/32 (50)	16/36 (44.44)	58/104 (55.77)
*Sciurotamias*	*Sciurotamias forresti*	0/1 (0)	0	0	0	0/1 (0)
*Soriculus*	*Soriculus leucops*	0	0	0	3/4 (75)	3/4 (75)
*Suncus*	*Suncus murinus*	1/2 (50)	5/18 (27.78)	8/10 (80)	3/5 (60)	17/35 (48.57)
	*Sorex minutes*	0	0	0	0/2 (0)	0/2 (0)
*Tupaia*	*Tupaia belangeri*	0/15 (0)	1/6 (16.67)	0/11 (0)	0/14 (0)	1/46 (2.17)
*Vernaya*	*Vernaya fulva*	0	1/1 (100)	0	0	1/1 (100)
Total	171/359 (47.63)	208/368 (56.52)	211/469 (44.99)	178/409 (43.52)	768/1605 (47.85)	

### 3.2 Associated characteristics of small mammals and environmental factor for *Bartonella* infection

Comparing *Bartonella* infection in small mammals across the different species, *E. mileyus* had the lowest *Bartonella* infection rate than that in *A. chevrieri*, *R. tanezumi*, *A. draco* (*p* < 0.001). Still, the other three species (*A. chevrieri*, *R. tanezumi*, *A. draco*) had no significant differences from one another. Small mammals that were male had higher infection rate than females (*p* < 0.05). Comparing the prevalence of *Bartonella* in small mammals from the three counties, the prevalence of *Bartonella* in small mammals from Lianghe county had the highest infection rate, followed by those in Yulong, the lowest infection was in Jianchuan (*p* < 0.001). Among the four seasons, *Bartonella* infection of small mammals in summer had the highest infection than that in other three seasons (*p* = 0.001). In addition, *Bartonella* infection in small mammals in woodland had the highest infection than that in bush and cultivation (*p* = 0.001). The relationship between the associated factors and the infection of *Bartonella* in small mammals is shown in Table 3.

**Table 3:** Factor analysis of *Bartonella* infection rates in wild small mammals collected from the three counties of western Yunnan Province.

Variables	Samples (*n* = 1,605)	Positive samples (*n* = 768)	Positive rate (%)	*p*-value
Species	<0.001
*Eothenomys mileyus*	305	115	37.7	
*Apodemus chevrieri*	471	255	54.14	
*Rattus tanezumi*	104	58	55.77	
*Apodemus draco*	157	84	53.5	
other	568	256	45.07	
Gender	0.046
Male	858	431	50.23	
Female	747	337	45.11	
Area	<0.001
Jianchuan	648	259	39.97	
Lianghe	407	229	56.27	
Yulong	550	280	50.91	
Season	0.001
Spring	359	171	47.63	
Summer	368	208	56.52	
Autumn	469	211	44.99	
Winter	409	178	43.52	
Landscape	0.001
Bush	36	11	30.56	
Cultivation	534	228	42.7	
Woodland	1,035	529	51.11	

### 3.3 Identification of *Bartonella* species in small mammals

Out of 768 positive samples (280 were in Yulong, 259 were in Jianchuan, and the rest were 229 in Lianghe county), 96 positive sample were randomly selected for further sequence analysis. 33 samples (YL-2-181, YL-3-202, JC-3-011, LH-1-012, et al.) shared more than 96% identity to *Bartonella tribocorum*, where LH-1-068 and LH-1-115 were homologous up to 100%, JC-3-051, LH-1-012, LH-1-079, LH-1-098, LH-1-103, LH-3-015, LH-3-088, LH-3-198, and LH-4-139 (9 samples) were also shared more than 98% identity with *Bartonella elizabethae*, LH-3-058 shared 96% with *B. tribocorum*, but shared a higher identity with *Bartonella rattimassiliensis* at 99.6%. 31 samples (YL-3-147, JC-1-076, LH-1-046 et al.) shared more than 96% identity to *Bartonella rochalimae*, where LH-3-206 were homologous up to 100%. 18 samples (YL-3-101, JC-2-035, LH-1-026, et al.) shared more than 99% identity to *B. japonica*. YL-3-230, LH-1-094, and YL-1-011 (3 samples) shared more than 98% identity to *Bartonella taylorii*, and LH-1-094 also shared 97.2% identity to *Bartonella sylvatica*. YL-1-094 shared 96.8% identity with *Bartonella grahamii*, but shared a higher identity with *B. washoensis* at 98%.

**Figure 3:**
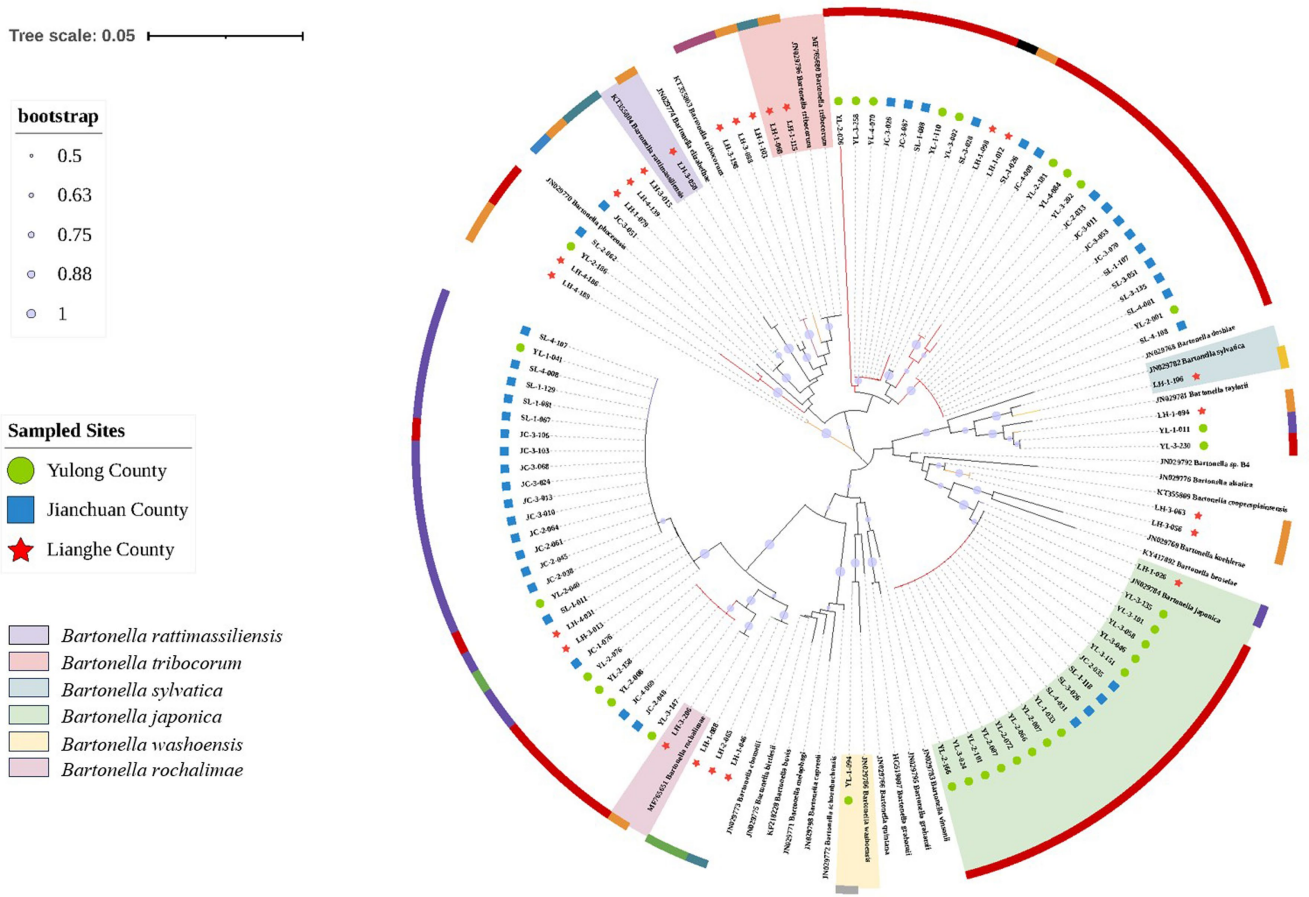
Neighbor-joining phylogenetic tree of *Bartonella* based on ssrA gene. Sequences from this study, green circle represented the positive samples in Yulong county, blue square represented the positive samples in Jianchuan county, red pentagram represented the positive samples in Lianghe county. The different colors of the outer circles represented the detection of *Bartonella* in different genera of small mammals: red represented *Apodemus* genera. Dark orange represented *Rattus* genera. Light orange represented *Mus* genera. Ligh green represented *Hylomys* genera. Dark green represented *Niviventer* genera. Blue represented *Anourosorex* genera. Purple represented *Eothenomys* genera. Dark pink represented *Suncus* genera. Gray represented *Dremomys* genera. Black represented *Crocidura* genera. The branch color was same represented the detection of *Bartonella* in different genera of small mammals. The different color shades represented the different *Bartonella* species.

The phylogenetic tree constructed with *ssrA* gene sequence homologies of 96 sample sequences and 27 reference sequences of *Bartonella* is shown in Figure 3. Phylogenetic analysis of sequences showed that the *Bartonella* strains detected in small mammals belonged to multiple clusters, including *B. rochalimae*, *Bartonella japonica*, *B. tribocorum*, *B. sylvatica*, *B. rattimassiliensis*, and *Bartonella washoensis*. *Bartonella* species in small mammals from Lianghe county belonged to five different strains, and it was more diverse than the strains of *Bartonella* identified from Yulong and Jianchuan counties, where only two and one strains were separately detected. The majority of sequences were ascribable to *B. japonica*. Among these positive samples, *A. chevrieri*, *E. mileyus*, and *A. draco* were detected with *Bartonella japonica*, *R. rattus* with *B. rattimassiliensis*, *Dremomys pernyi* with *B. washoensis*, *M. Pahari* with *B. sylvatica*, *N. fulvescens* with *B. tribocorum*, in addition, *R. tanezumi* was detected with both *B. tribocorum* and *B. rochalimae*. Sequence details from this study are shown in Supplementary Tables S1, S2.

## 4 Discussion

*Bartonella* has gained recognition as an emerging zoonotic pathogen. It has been detected in various small mammals in China, particular exhibiting a high infection rate in the northern and southwestern regions (23). In Yunnan Province, *Bartonella* infection in small mammals has been studied across diverse climates and habitats. Moreover, the presence of *Bartonella* natural foci in northwestern Yunnan has already been confirmed, indicating the remarkable adaptability of *Bartonella* in various small mammals by different geographic regions (20). Despite these advancements, however, a critical gap remains in our understand of the epidemiology and species diversity of *Bartonella* in nature. Thus, it is of great important need for systematic and comprehensive investigations into the epidemiological and ecological characteristics of *Bartonella* infection in small mammals in different geographic areas. In this study, 1,605 small mammals were tested. The overall prevalence of *Bartonella* was 47.85%, *B. rochalimae*, *B. japonica*, *B. tribocorum*, *B. washoensis*, *B. sylvatica*, and *B. rattimassiliensis* were the main species. The *Bartonella* infection rate in small mammals was aligned with previous studies in Yunnan, and the infection rate exhibited a range of 15.84% to 55.91% in the small mammal population (17–19, 24). However, the infection rate in small mammals was higher than that of 37.41% in Shanxi province (13), 30.1%–38.61% in Qinghai province (11, 12), but slightly lower than that of 59.09% in Tibet (25), this may be due to the influence of sampling sites, captured small mammals species, or differences in detection methods. Additionally, in Lianghe county, a notably high prevalence of *Bartonella* infection was detected, reaching 56.27% in small mammals. Interestingly, it is worth noting that one previous study in Lianghe county, which detected a limited set of species, including *R. tanezumi*, *Hylomys suillus*, and *Berylmys manipulus*, did not detect *Bartonella* infections (19). In contrast, the present study expanded its scope by testing *Bartonella* in a broader ranges of small mammal species, encompassing a total of 30 small mammal species. This broader species detection suggests an increase likelihood of detecting *Bartonella* infection in small mammals, enhancing the potential influence of species richness on *Bartonella* detection rates.

A variety of small mammals can carry *Bartonella* in nature. Generally, small mammals are the main hosts of *Bartonella*. Diverse dominant small mammal species in different areas would lead to various primary hosts of *Bartonella*. The infection rate of *Bartonella* among small mammals in Vietnam was 14.9%, and the infection rate of *R. tanezumi* was the highest (49.2%), followed by *Rattus norvegicus* (20.7%) (26). In this study, the prevalence of *Bartonella* was highest in *N. fulvescens* (85.37%, 35/41), followed by *A. latronum*. Small mammals that survive in different areas determine the possibility of species carrying *Bartonella*. In this study, a slightly higher *Bartonella* infection was detected in male small mammals compared to females. This finding was similar to the previous study and could be attributed to the more vigorous activity of male small mammals, which can elevate their chances for exposure to pathogens and enhance the potential roles serving as a host for transmission to other small mammals (27).

Yulong and Jianchuan counties have similar dominant hosts and environments. Still, the rates of *Bartonella* infection in the two areas were different, and this could be due to variations in the number of dominant small mammal species infected with *Bartonella*, suggesting differences in how small mammals adapt to the pathogens in similar environments. In addition, the dominant small mammal species in Lianghe county also differed from those in Yulong and Jianchuan counties, with higher infection rates in *R. tanezumi*. This finding was in line with previous studies that showed *R. tanezumi* was the main host for *Bartonella* (7, 16, 17). In addition, *Apodemus* and *Eothenomys* genera had higher infection rates of *Bartonella* in Yulong and Jianchuan counties, and this finding also was in line with previous studies showed that *Apodemus* and *Eothenomys* genera were the main genera for *Bartonella* in small mammals (28, 29). The genera of *Rattus*, *Apodemus*, and *Eothenomys* are Yunnan’s the dominant small mammal species. Thus, it holds significant importance to maintain sustained surveillance of the genera of *Rattus*, *Apodemus*, and *Eothenomys* in small mammals. This proactive approach is crucial for preventing *Bartonella* infections from spilling over from natural reservoirs to humans, particularly when infected small mammal populations surpass a critical threshold.

The prevalence of *Bartonella* in small mammals had the highest rate in summer than in the other three seasons. This result was consistence with previous study, which showed that *Bartonella* infection of small mammals was higher in summer in Vietnam, Laos, and Thailand, suggesting that *Bartonella* infection status could be affected by the dynamics distribution of hosts, the abundance of vector parasite on small mammals in different seasons (30). It has been reported that temperature has a strong influence on the growth of fleas, indirectly affecting the infected flea transmission when seasons change, therefore indirectly affecting the spread of *Bartonella* (31, 32). *Bartonella* infection in small mammals inhabit in woodland and cultivation was higher, which was similar to previous study, such as a higher *Bartonella* infection rate in small mammals inhabited in forest and farmland in Shangdang Basin, China (13), indicating that the higher risk infection of *Bartonella* when people go to the woodland or farmland, especially travelers are more likely closer to the natural environments.

Based on *ssrA* gene, 768 positive samples were detected in small mammals, and the sequencing of positive samples was generated by conventional PCR, 96 samples were randomly selected and sequenced for molecular analysis. Six *Bartonella* species were obtained from small mammals based on the neighbor-joining phylogenetic tree, including *B. rochalimae*, *B. japonica*, *B. tribocorum*, *B. sylvatica*, *B. rattimassiliensis*, and *B. washoensis*. In addition, *B. tribocorum* could detect in *N. fulvescens* and *R. tanezumi*. *B. japonica* could detect in *A. chevrieri*, *E. mileyus*, and *A. draco*. Other strains of *Bartonella* were detected in one small mammal species, suggesting that highly adaptability and species diversity of *Bartonella* in small mammals, thus, long-term surveillance of *Bartonella* infection in small mammal is necessary to prevent the transmission of pathogens from nature to humans by infected small mammals. Moreover, *R. tanezumi* can detect *B. rochalimae* expect *B. tribocorum*, the two *R. tanezumi* in the same landscape and county were not completely the same, one *R. tanezumi* infected *B. tribocorum* were male captured in spring, but another *R. tanezumi* infected *B. rochalimae* were female captured in autumn, indicating that the diversity of same small mammal species in similar environments, and high adaptability of *Bartonella* to host.

This study has some limitations. First, there are twenty counties in western Yunnan Province, and this study only selected three counties as the sample sites. However, a large number of small mammals were captured in each county (550 were in Yulong County, 648 were in Jianchuan County, and 407 were in Lianghe County), which could improve the representativeness of the composition and distribution of small mammals in western Yunnan Province. Second, only 96 out of the 768 positive samples were sequenced due to inadequate research project funding, but the randomly selected samples included different counties and landscapes whenever possible. The above selection criteria could be biased, but the sequencing results of positive samples should be as representative as possible. As a result, there might be an insufficient number of *Bartonella* species identified in wild small mammals.

## 5 Conclusion

A high prevalence of *Bartonella* was detected in Yulong, Jianchuan, and Lianghe counties of western Yunnan Province. Six *Bartonella* species (*B. rochalimae*, *B. japonica*, *B. tribocorum*, *B. sylvatica*, *B. rattimassiliensis*, and *B. washoensis*) were obtained, with *B. japonica* being the main species. The rates of *Bartonella* infection were influenced by small mammal species, the gender of small mammals, geographical areas, seasons, and landscapes. Therefore, ongoing surveillance for *Bartonella* infection in small mammals is crucial, and efforts to identify *Bartonella* species should be maximized to prevent human infections caused by infected small mammals.

## Data availability statement

The original contributions presented in the study are included in the article/Supplementary material, further inquiries can be directed to the corresponding author.

## Ethics statement

The animal study was approved by Medical Ethics Committee of Dali University. The study was conducted in accordance with the local legislation and institutional requirements.

## Author contributions

Y-YL: Data curation, Formal analysis, Software, Visualization, Writing – original draft, Writing – review & editing. DY: Data curation, Formal analysis, Methodology, Software, Visualization, Writing – original draft. H-ZZ: Data curation, Formal analysis, Methodology, Writing – original draft. Z-XL: Investigation, Writing – original draft. R-DH: Investigation, Methodology, Writing – original draft. MH: Investigation, Writing – original draft. ZQ-A: Methodology, Writing – original draft. J-JZ: Methodology, Writing – original draft. J-XY: Conceptualization, Funding acquisition, Investigation, Project administration, Resources, Supervision, Validation, Writing – review & editing.

## Glossary

**Table:** 

qPCR	Real-time quantitative polymerase chain reaction
DNA	Deoxyribonucleic acid
Small mammal species	
*A. chevrieri*	*Apodemus chevrieri*
*A. draco*	*Apodemus draco*
*C. dracula*	*Crocidura dracula*
*E. mileyus*	*Eothenomys mileyus*
*M. pahari*	*Mus pahari*
*N. andersoni*	*Niviventer andersoni*
*N. confucianus*	*Niviventer confucianus*
*N. fulvescens*	*Niviventer fulvescens*
*R. tanezumi*	*Rattus tanezumi*
*R. rattus*	*Rattus rattus*
Bartonella species	
*B. rochalimae*	*Bartonella rochalimae*
*B. japonica*	*Bartonella japonica*
*B. tribocorum*	*Bartonella tribocorum*
*B. sylvatica*	*Bartonella sylvatica*
*B. rattimassiliensis*	*Bartonella rattimassiliensis*
*B. washoensis*	*Bartonella washoensis*
*B. elizabethae*	*Bartonella elizabethae*
*B. elizabethae*	*Bartonella elizabethae*
*B. taylorii*	*Bartonella taylorii*
*B. grahamii*	*Bartonella grahamii*

## Footnotes

^1^ https://blast.ncbi.nlm.nih.gov/Blast.cgi
