# Supplementary material for: Molecular detection of Bartonella species in wild small mammals in western Yunnan Province, China
Source: Front Vet Sci. 2023 Nov 21;10:1301316. doi: 10.3389/fvets.2023.1301316 (PMC10703294; doi:10.3389/fvets.2023.1301316)
Supplement: Supplementary file 1 [file Table_1.DOCX]

Supplementary Table 1

Supplementary Table 1 *Bartonella* genotypes of the infected wild small mammals in western Yunnan Province

| Areas | Samples^*^ | Season | Small mammals | Genera | Families | Orders | Genotypes |
| --- | --- | --- | --- | --- | --- | --- | --- |
| Jianchuan | JC-2-035 | Summer | *Apodemus chevrieri* | *Apodemus* | *Murinae* | *Rodentia* | *Bartonella japonica* |
| Jianchuan | SL-1-118 | Spring | *Apodemus chevrieri* | *Apodemus* | *Murinae* | *Rodentia* | *Bartonella japonica* |
| Jianchuan | SL-3-026 | Autumn | *Apodemus chevrieri* | *Apodemus* | *Murinae* | *Rodentia* | *Bartonella japonica* |
| Jianchuan | SL-4-031 | Winter | *Apodemus chevrieri* | *Apodemus* | *Murinae* | *Rodentia* | *Bartonella japonica* |
| Jianchuan | JC-1-076 | Spring | *Eothenomys mileyus* | *Eothenomys* | *Cricetidae* | *Rodentia* | Unspecified |
| Jianchuan | JC-2-033 | Summer | *Apodemus chevrieri* | *Apodemus* | *Murinae* | *Rodentia* | Unspecified |
| Jianchuan | JC-2-038 | Summer | *Eothenomys melanogaster* | *Eothenomys* | *Cricetidae* | *Rodentia* | Unspecified |
| Jianchuan | JC-2-045 | Summer | *Eothenomys mileyus* | *Eothenomys* | *Cricetidae* | *Rodentia* | Unspecified |
| Jianchuan | JC-2-048 | Summer | *Apodemus chevrieri* | *Apodemus* | *Murinae* | *Rodentia* | Unspecified |
| Jianchuan | JC-2-061 | Summer | *Eothenomys mileyus* | *Eothenomys* | *Cricetidae* | *Rodentia* | Unspecified |
| Jianchuan | JC-2-064 | Summer | *Eothenomys mileyus* | *Eothenomys* | *Cricetidae* | *Rodentia* | Unspecified |
| Jianchuan | JC-3-010 | Autumn | *Eothenomys mileyus* | *Eothenomys* | *Cricetidae* | *Rodentia* | Unspecified |
| Jianchuan | JC-3-011 | Autumn | *Apodemus chevrieri* | *Apodemus* | *Murinae* | *Rodentia* | Unspecified |
| Jianchuan | JC-3-013 | Autumn | *Eothenomys mileyus* | *Eothenomys* | *Cricetidae* | *Rodentia* | Unspecified |
| Jianchuan | JC-3-024 | Autumn | *Eothenomys mileyus* | *Eothenomys* | *Cricetidae* | *Rodentia* | Unspecified |
| Jianchuan | JC-3-026 | Autumn | *Apodemus chevrieri* | *Apodemus* | *Murinae* | *Rodentia* | Unspecified |
| Jianchuan | JC-3-051 | Autumn | *Anourosorex squamipes* | *Anourosorex* | *Soricidae* | *Insectivora* | Unspecified |

Supplementary Table 1 *Bartonella* genotypes of the infected wild small mammals in western Yunnan Province (Continued)

| Areas | Samples^*^ | Season | Small mammals | Genera | Families | Orders | Genotypes |
| --- | --- | --- | --- | --- | --- | --- | --- |
| Jianchuan | JC-3-053 | Autumn | *Apodemus chevrieri* | *Apodemus* | *Murinae* | *Rodentia* | Unspecified |
| Jianchuan | JC-3-068 | Autumn | *Eothenomys mileyus* | *Eothenomys* | *Cricetidae* | *Rodentia* | Unspecified |
| Jianchuan | JC-3-070 | Autumn | *Apodemus chevrieri* | *Apodemus* | *Murinae* | *Rodentia* | Unspecified |
| Jianchuan | JC-3-087 | Autumn | *Apodemus chevrieri* | *Apodemus* | *Murinae* | *Rodentia* | Unspecified |
| Jianchuan | JC-3-103 | Autumn | *Eothenomys mileyus* | *Eothenomys* | *Cricetidae* | *Rodentia* | Unspecified |
| Jianchuan | JC-3-106 | Autumn | *Apodemus chevrieri* | *Apodemus* | *Murinae* | *Rodentia* | Unspecified |
| Jianchuan | JC-4-009 | Winter | *Apodemus chevrieri* | *Apodemus* | *Murinae* | *Rodentia* | Unspecified |
| Jianchuan | JC-4-069 | Winter | *Apodemus chevrieri* | *Apodemus* | *Murinae* | *Rodentia* | Unspecified |
| Jianchuan | SL-1-011 | Spring | *Eothenomys mileyus* | *Eothenomys* | *Cricetidae* | *Rodentia* | Unspecified |
| Jianchuan | SL-1-026 | Spring | *Apodemus draco* | *Apodemus* | *Murinae* | *Rodentia* | Unspecified |
| Jianchuan | SL-1-067 | Spring | *Eothenomys mileyus* | *Eothenomys* | *Cricetidae* | *Rodentia* | Unspecified |
| Jianchuan | SL-1-081 | Spring | *Eothenomys mileyus* | *Eothenomys* | *Cricetidae* | *Rodentia* | Unspecified |
| Jianchuan | SL-1-088 | Spring | *Apodemus chevrieri* | *Apodemus* | *Murinae* | *Rodentia* | Unspecified |
| Jianchuan | SL-1-107 | Spring | *Apodemus chevrieri* | *Apodemus* | *Murinae* | *Rodentia* | Unspecified |
| Jianchuan | SL-1-129 | Spring | *Eothenomys mileyus* | *Eothenomys* | *Cricetidae* | *Rodentia* | Unspecified |
| Jianchuan | SL-2-062 | Summer | *Apodemus chevrieri* | *Apodemus* | *Murinae* | *Rodentia* | Unspecified |
| Jianchuan | SL-3-028 | Autumn | *Apodemus chevrieri* | *Apodemus* | *Murinae* | *Rodentia* | Unspecified |
| Jianchuan | SL-3-051 | Autumn | *Apodemus draco* | *Apodemus* | *Murinae* | *Rodentia* | Unspecified |

Supplementary Table 1 *Bartonella* genotypes of the infected wild small mammals in western Yunnan Province (Continued)

| Areas | Samples^*^ | Season | Small mammals | Genera | Families | Orders | Genotypes |
| --- | --- | --- | --- | --- | --- | --- | --- |
| Jianchuan | SL-3-135 | Autumn | *Apodemus chevrieri* | *Apodemus* | *Murinae* | *Rodentia* | Unspecified |
| Jianchuan | SL-4-008 | Winter | *Eothenomys mileyus* | *Eothenomys* | *Cricetidae* | *Rodentia* | Unspecified |
| Jianchuan | SL-4-081 | Winter | *Apodemus chevrieri* | *Apodemus* | *Murinae* | *Rodentia* | Unspecified |
| Jianchuan | SL-4-107 | Winter | *Eothenomys mileyus* | *Eothenomys* | *Cricetidae* | *Rodentia* | Unspecified |
| Jianchuan | SL-4-108 | Winter | *Apodemus chevrieri* | *Apodemus* | *Murinae* | *Rodentia* | Unspecified |
| Lianghe | LH-1-115 | Spring | *Rattus tanezumi* | *Rattus* | *Murinae* | *Rodentia* | *Bartonella tribocorum* |
| Lianghe | LH-1-068 | Spring | *Niviventer fulvescens* | *Niviventer* | *Murinae* | *Rodentia* | *Bartonella tribocorum* |
| Lianghe | LH-1-106 | Spring | *Mus pahari* | *Mus* | *Murinae* | *Rodentia* | *Bartonella sylvatica* |
| Lianghe | LH-3-058 | Autumn | *Rattus rattus* | *Rattus* | *Murinae* | *Rodentia* | *Bartonella rattimassiliensis* |
| Lianghe | LH-3-206 | Autumn | *Rattus tanezumi* | *Rattus* | *Murinae* | *Rodentia* | *Bartonella rochalimae* |
| Lianghe | LH-1-026 | Spring | *Eothenomys mileyus* | *Eothenomys* | *Cricetidae* | *Rodentia* | *Bartonella japonica* |
| Lianghe | LH-1-012 | Spring | *Rattus rattus* | *Rattus* | *Murinae* | *Rodentia* | Unspecified |
| Lianghe | LH-1-046 | Spring | *Niviventer fulvescens* | *Niviventer* | *Murinae* | *Rodentia* | Unspecified |
| Lianghe | LH-1-079 | Spring | *Rattus tanezumi* | *Rattus* | *Murinae* | *Rodentia* | Unspecified |
| Lianghe | LH-1-088 | Spring | *Hylomys suillus* | *Hylomys* | *Erinaceidae* | *Insectivora* | Unspecified |
| Lianghe | LH-1-094 | Spring | *Rattus tanezumi* | *Rattus* | *Murinae* | *Rodentia* | Unspecified |
| Lianghe | LH-1-098 | Spring | *Crocidura attenuate* | *Crocidura* | *Soricidae* | *Insectivora* | Unspecified |
| Lianghe | LH-1-103 | Spring | *Rattus tanezumi* | *Rattus* | *Murinae* | *Rodentia* | Unspecified |

Supplementary Table 1 *Bartonella* genotypes of the infected wild small mammals in western Yunnan Province (Continued)

| Areas | Samples^*^ | Season | Small mammals | Genera | Families | Orders | Genotypes |
| --- | --- | --- | --- | --- | --- | --- | --- |
| Lianghe | LH-2-065 | Summer | *Hylomys suillus* | *Hylomys* | *Erinaceidae* | *Insectivora* | Unspecified |
| Lianghe | LH-3-013 | Autumn | *Eothenomys eleusis* | *Eothenomys* | *Cricetidae* | *Rodentia* | Unspecified |
| Lianghe | LH-3-015 | Autumn | *Niviventer fulvescens* | *Niviventer* | *Murinae* | *Rodentia* | Unspecified |
| Lianghe | LH-3-056 | Autumn | *Rattus rattus* | *Rattus* | *Murinae* | *Rodentia* | Unspecified |
| Lianghe | LH-3-063 | Autumn | *Rattus rattus* | *Rattus* | *Murinae* | *Rodentia* | Unspecified |
| Lianghe | LH-3-088 | Autumn | *Suncus murinus* | *Suncus* | *Soricidae* | *Insectivora* | Unspecified |
| Lianghe | LH-3-198 | Autumn | *Suncus murinus* | *Suncus* | *Soricidae* | *Insectivora* | Unspecified |
| Lianghe | LH-4-031 | Winter | *Hylomys suillus* | *Hylomys* | *Erinaceidae* | *Insectivora* | Unspecified |
| Lianghe | LH-4-139 | Winter | *Niviventer fulvescens* | *Niviventer* | *Murinae* | *Rodentia* | Unspecified |
| Lianghe | LH-4-186 | Winter | *Rattus tanezumi* | *Rattus* | *Murinae* | *Rodentia* | Unspecified |
| Lianghe | LH-4-189 | Winter | *Rattus tanezumi* | *Rattus* | *Murinae* | *Rodentia* | Unspecified |
| Yulong | YL-1-094 | Spring | *Dremomys pernyi* | *Dremomys* | *Sciuridae* | *Rodentia* | *Bartonella washoensis* |
| Yulong | YL-1-033 | Spring | *Apodemus chevrieri* | *Apodemus* | *Murinae* | *Rodentia* | *Bartonella japonica* |
| Yulong | YL-2-007 | Summer | *Apodemus chevrieri* | *Apodemus* | *Murinae* | *Rodentia* | *Bartonella japonica* |
| Yulong | YL-2-066 | Summer | *Apodemus chevrieri* | *Apodemus* | *Murinae* | *Rodentia* | *Bartonella japonica* |
| Yulong | YL-2-072 | Summer | *Apodemus chevrieri* | *Apodemus* | *Murinae* | *Rodentia* | *Bartonella japonica* |
| Yulong | YL-2-087 | Summer | *Apodemus chevrieri* | *Apodemus* | *Murinae* | *Rodentia* | *Bartonella japonica* |
| Yulong | YL-2-101 | Summer | *Apodemus draco* | *Apodemus* | *Murinae* | *Rodentia* | *Bartonella japonica* |

Supplementary Table 1 *Bartonella* genotypes of the infected wild small mammals in western Yunnan Province (Continued)

| Areas | Samples^*^ | Season | Small mammals | Genera | Families | Orders | Genotypes |
| --- | --- | --- | --- | --- | --- | --- | --- |
| Yulong | YL-2-166 | Summer | *Apodemus draco* | *Apodemus* | *Murinae* | *Rodentia* | *Bartonella japonica* |
| Yulong | YL-3-024 | Autumn | *Apodemus chevrieri* | *Apodemus* | *Murinae* | *Rodentia* | *Bartonella japonica* |
| Yulong | YL-3-046 | Autumn | *Apodemus chevrieri* | *Apodemus* | *Murinae* | *Rodentia* | *Bartonella japonica* |
| Yulong | YL-3-058 | Autumn | *Apodemus chevrieri* | *Apodemus* | *Murinae* | *Rodentia* | *Bartonella japonica* |
| Yulong | YL-3-101 | Autumn | *Apodemus chevrieri* | *Apodemus* | *Murinae* | *Rodentia* | *Bartonella japonica* |
| Yulong | YL-3-135 | Autumn | *Apodemus chevrieri* | *Apodemus* | *Murinae* | *Rodentia* | *Bartonella japonica* |
| Yulong | YL-3-151 | Autumn | *Apodemus chevrieri* | *Apodemus* | *Murinae* | *Rodentia* | *Bartonella japonica* |
| Yulong | YL-1-011 | Spring | *Eothenomys proditor* | *Eothenomys* | *Cricetidae* | *Rodentia* | Unspecified |
| Yulong | YL-1-041 | Spring | *Eothenomys mileyus* | *Eothenomys* | *Cricetidae* | *Rodentia* | Unspecified |
| Yulong | YL-1-110 | Spring | *Apodemus chevrieri* | *Apodemus* | *Murinae* | *Rodentia* | Unspecified |
| Yulong | YL-2-001 | Summer | *Apodemus draco* | *Apodemus* | *Murinae* | *Rodentia* | Unspecified |
| Yulong | YL-2-008 | Summer | *Apodemus chevrieri* | *Apodemus* | *Murinae* | *Rodentia* | Unspecified |
| Yulong | YL-2-026 | Summer | *Apodemus draco* | *Apodemus* | *Murinae* | *Rodentia* | Unspecified |
| Yulong | YL-2-040 | Summer | *Apodemus chevrieri* | *Apodemus* | *Murinae* | *Rodentia* | Unspecified |
| Yulong | YL-2-076 | Summer | *Apodemus chevrieri* | *Apodemus* | *Murinae* | *Rodentia* | Unspecified |
| Yulong | YL-2-158 | Summer | *Apodemus draco* | *Apodemus* | *Murinae* | *Rodentia* | Unspecified |
| Yulong | YL-2-181 | Summer | *Apodemus draco* | *Apodemus* | *Murinae* | *Rodentia* | Unspecified |
| Yulong | YL-2-186 | Summer | *Apodemus latronum* | *Apodemus* | *Murinae* | *Rodentia* | Unspecified |

Supplementary Table 1 *Bartonella* genotypes of the infected wild small mammals in western Yunnan Province (Continued)

| Areas | Samples^*^ | Season | Small mammals | Genera | Families | Orders | Genotypes |
| --- | --- | --- | --- | --- | --- | --- | --- |
| Yulong | YL-3-002 | Autumn | *Apodemus chevrieri* | *Apodemus* | *Murinae* | *Rodentia* | Unspecified |
| Yulong | YL-3-147 | Autumn | *Apodemus chevrieri* | *Apodemus* | *Murinae* | *Rodentia* | Unspecified |
| Yulong | YL-3-202 | Autumn | *Apodemus chevrieri* | *Apodemus* | *Murinae* | *Rodentia* | Unspecified |
| Yulong | YL-3-230 | Autumn | *Apodemus chevrieri* | *Apodemus* | *Murinae* | *Rodentia* | Unspecified |
| Yulong | YL-3-258 | Autumn | *Apodemus chevrieri* | *Apodemus* | *Murinae* | *Rodentia* | Unspecified |
| Yulong | YL-4-070 | Winter | *Apodemus chevrieri* | *Apodemus* | *Murinae* | *Rodentia* | Unspecified |
| Yulong | YL-4-084 | Winter | *Apodemus draco* | *Apodemus* | *Murinae* | *Rodentia* | Unspecified |

^*^Notes: LH: Lianghe county; JC and SL: Jianchuan county; YL: Yulong county.

1: Spring; 2: Summer; 3: Autumn; 4: Winter
