# Supplementary material for: Molecular detection of Bartonella species in wild small mammals in western Yunnan Province, China
Source: Front Vet Sci. 2023 Nov 21;10:1301316. doi: 10.3389/fvets.2023.1301316 (PMC10703294; doi:10.3389/fvets.2023.1301316)
Supplement: Supplementary file 2 [file Table_2.DOCX]

Table S2 *Bartonella* species reference strains based on *ssrA* gene from GenBank used in this study

| Species of strain | Source | No. Genbank | Geographic location | Time | DNA size(bp) |
| --- | --- | --- | --- | --- | --- |
| *Bartonella tribocorum* | Human | JN029796 | USA | 2011 | 253 |
| *Bartonella tribocorum* | Rodent | MF765680 | China | 2017 | 301 |
| *Bartonella japonica* | Human | JN029784 | USA | 2011 | 252 |
| *Bartonella koehlerae* | Human | JN029769 | USA | 2011 | 252 |
| *Bartonella henselae* | Flea | KY417892 | New South Wales | 2016 | 252 |
| *Bartonella coopersplainsensis* | Rodent | KT355809 | Thailand | 2015 | 252 |
| *Bartonella alsatica* | Human | JN029776 | USA | 2011 | 254 |
| *Bartonella sp B4* | Human | JN029792 | USA | 2011 | 247 |
| *Bartonella doshiae* | Human | JN029768 | USA | 2011 | 253 |
| *Bartonella silvatica* | Human | JN029782 | USA | 2011 | 253 |
| *Bartonella taylori* | Human | JN029781 | USA | 2011 | 253 |
| *Bartonella grahamii* | Human | JN029795 | USA | 2011 | 253 |
| *Bartonella grahamii* | - | HG519007 | USA | 2013 | 318 |
| *Bartonella vinsonii* | Human | JN029783 | USA | 2011 | 251 |
| *Bartonella quintana* | Human | JN029766 | USA | 2011 | 249 |
| *Bartonella washoensis* | Human | JN029786 | USA | 2011 | 253 |
| *Bartonella birtlesii* | Human | JN029775 | USA | 2011 | 251 |
| *Bartonella chomelii* | Human | JN029773 | USA | 2011 | 251 |
| *Bartonella bovis* | Bovine | KF218228 | Thailand | 2013 | 287 |
| *Bartonella melophagi* | Human | JN029771 | USA | 2011 | 252 |
| *Bartonella capreoli* | Human | JN029798 | USA | 2011 | 251 |
| *Bartonella schoenbuchensis* | Human | JN029772 | USA | 2011 | 251 |
| *Bartonella rochalimae* | Rodent | MF765651 | China | 2017 | 301 |
| *Bartonella tribocorum* | Rodent | KT355803 | Thailand | 2015 | 253 |
| *Bartonella elizabethae* | Human | JN029774 | USA | 2011 | 253 |
| *Bartonella rattimassiliensis* | Rodent | KT355804 | Thailand | 2015 | 253 |
| *Bartonella phoceensis* | Human | JN029770 | USA | 2011 | 252 |
