# Supplementary figures and images for: Molecular detection of Bartonella species in wild small mammals in western Yunnan Province, China
Source: Front Vet Sci. 2023 Nov 21;10:1301316. doi: 10.3389/fvets.2023.1301316 (PMC10703294; doi:10.3389/fvets.2023.1301316)

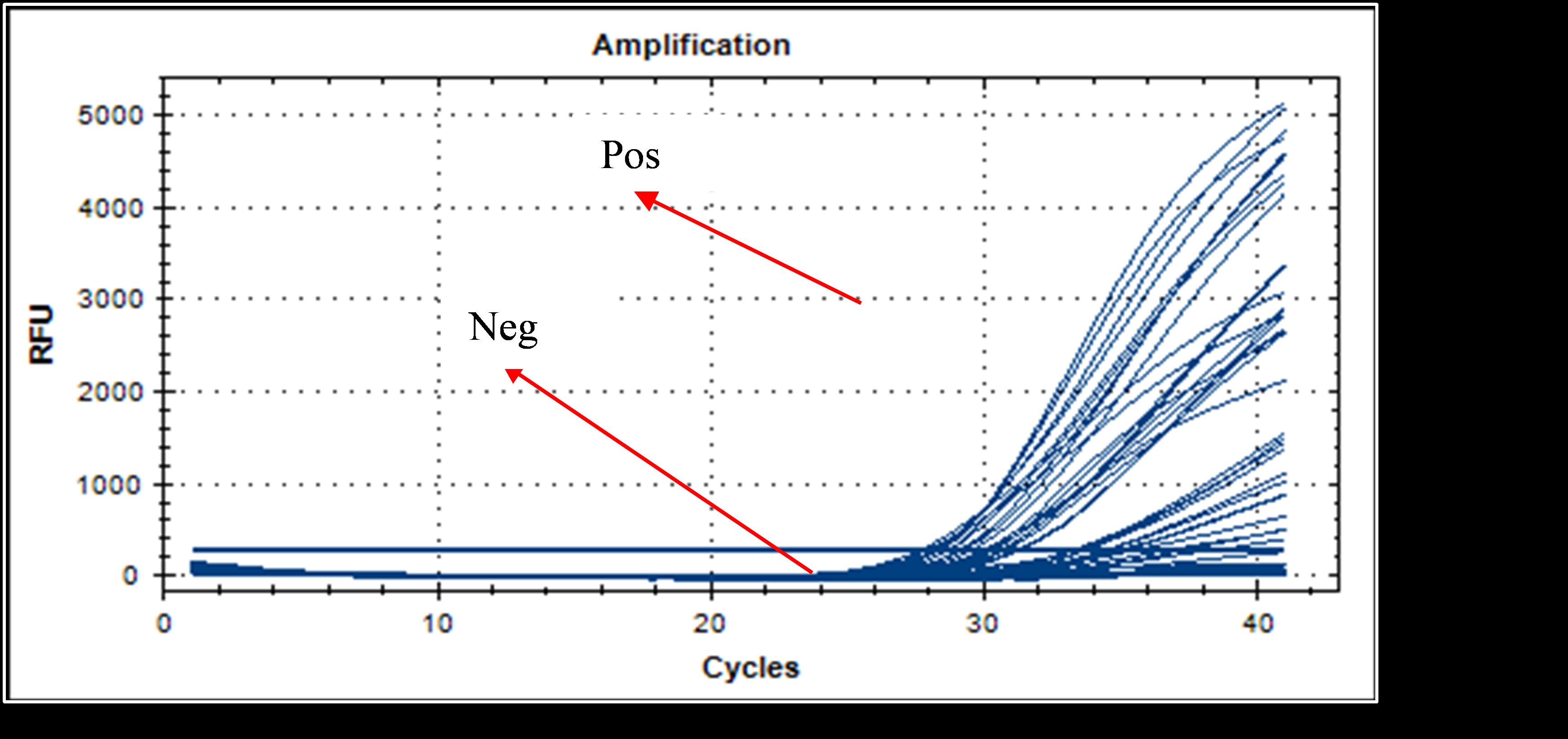

Supplement: Supplementary file 3 [file Image_1.JPEG]

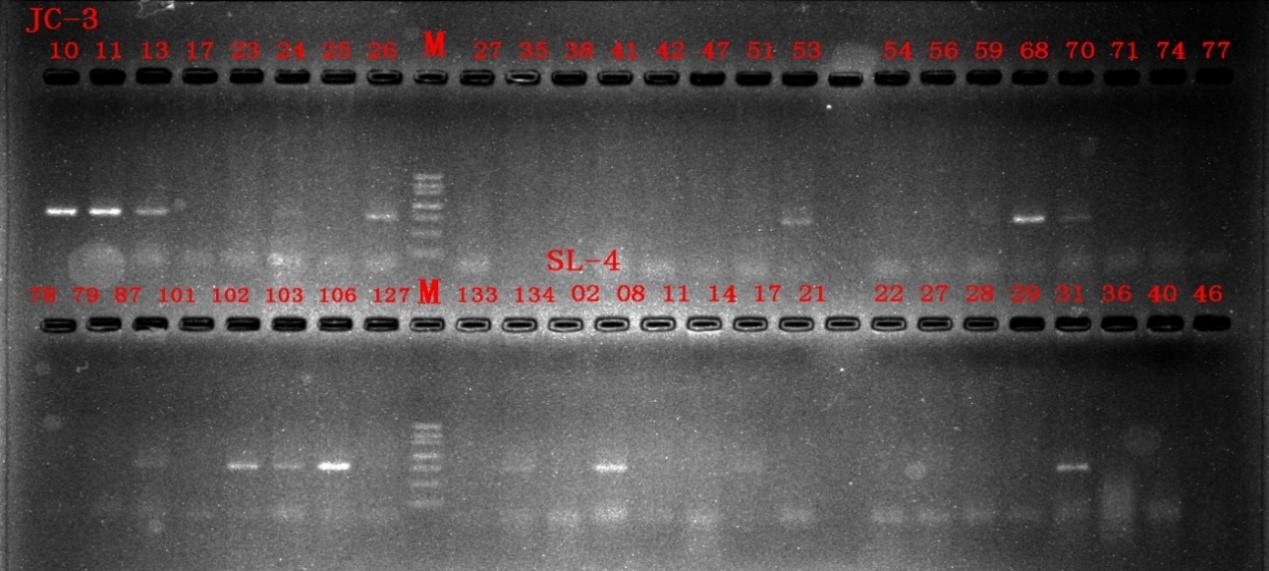

Supplement: Supplementary file 4 [file Image_2.JPEG]
